# Investigation of volatile sulfur compound level and halitosis in patients with gingivitis and periodontitis

**DOI:** 10.1038/s41598-023-40391-3

**Published:** 2023-08-14

**Authors:** Yeon-Hee Lee, Seung-Il Shin, Ji-Youn Hong

**Affiliations:** 1 Department of Orofacial Pain and Oral Medicine, Kyung Hee University, Kyung Hee University Dental Hospital, #613 Hoegi-dong, Dongdaemun-gu, Seoul, 02447 Korea; 2 Department of Periodontology, Periodontal-Implant Clinical Research Institute, School of Dentistry, Kyung Hee University, 26, Kyungheedae-ro, Dongdaemun-gu, Seoul, 02447 Korea

**Keywords:** Diseases, Medical research, Risk factors, Signs and symptoms

## Abstract

This study aimed to measure the levels of volatile sulfur compounds and investigate the occurrence of halitosis in patients with gingivitis and periodontitis. Additionally, the incidence rates of gingivitis and periodontitis in patients with halitosis were investigated. Through various statistical analyses, we attempted to determine the relationship between periodontal disease and halitosis. One-hundred-and-four participants (52 females and 52 males, mean age: 46.49 ± 16.03 years) were enrolled in this cross-sectional study, comprising 33 healthy controls, 43 patients with gingivitis, and 28 patients with periodontitis. Gas chromatography was used to measure hydrogen sulfide (H_2_S) and methyl mercaptan (CH_3_SH), which are representative VSCs. The VSC cut-off values for diagnosing halitosis were 65.79 ppb for women and 79.94 ppb for men. Total VSC level was significantly higher in the gingivitis than the healthy control group (186.72 ± 374.83 ppb vs. 19.80 ± 40.19 ppb, p = 0.035). There was no significant difference between the gingivitis and periodontitis (153.79 ± 278.51 ppb) groups. H_2_S level was significantly higher in the gingivitis (100.51 ± 183.69 ppb) and periodontitis (91.57 ± 132.06 ppb) groups than in healthy controls (14.97 ± 31.22 ppb), and CH_3_SH level was significantly higher in gingivitis group (29.31 ± 59.16 ppb) than in the healthy control (5.73 ± 14.10 ppb) (all p < 0.05). Halitosis was found in 3% of healthy controls and 39.5% and 42.9% of patients with gingivitis and periodontitis patients, respectively, making it significantly higher in the gingivitis and periodontitis groups than the healthy controls (p = 0.005). Conversely, among participants with halitosis, 53.1% had gingivitis, 37.5% had periodontitis, and 90.6 incidence had periodontal disease. Multivariate logistic regression analysis to predict the presence of halitosis, found periodontal disease was a significant predictor of halitosis (OR = 3.607, 95% CI 1.023–12.718, p = 0.046). Considering area under curve value for halitosis, the cut-off value of healthy control (H_2_S:61.5 ppb, CH_3_SH:3.5 ppb), gingivitis (H_2_S:50.0 ppb, CH_3_SH:6 ppb), and periodontitis (H_2_S:62.0 ppb, CH_3_SH:3.5 ppb) were (all p < 0.05). Our results emphasize the close and strong relationship between periodontal disease and halitosis through human clinical evidence based on the high co-occurrence rate of mutual diseases. Additionally, the presence of periodontal disease increased the probability of halitosis by 3.607 times. These results suggest that H_2_S can be used as a biomarker of halitosis in patients with periodontal disease.

## Introduction

Halitosis, oral malodor, and bad breath are all terms used to refer to noxious odors from the mouth when breathing or talking. Bad breath is common at all times and places, and 31.8% of people worldwide have halitosis^1^. The etiology of oral malodor is complex. Sources of halitosis are largely divided into intraoral and extraoral causes^2^. However, clinicians and researchers have decided to use the term oral malodor because up to 90% of the causes originate from the oral cavity^3^. The most common oral spaces where halitosis occurs include the posterior tongue dorsum; various periodontal tissues such as the gingival sulcus, pathological pockets, and interdental spaces; and defects around dental restorations, dental carious lesions, and poorly maintained dentures, which become a niche for microbiota to live^4^. Other pathological conditions from oral sources that may contribute to or causes halitosis include periodontal diseases, xerostomia, candidiasis, abscesses, oral mucositis, and oral cancers^5^.

Halitosis is mainly caused by the microbial degradation of desquamated human epithelial cells, blood cell debris, dental plaque, proteins in saliva and blood, and sulfur-containing amino acids present in the tongue coating^6^. Volatile sulfur compounds (VSCs) are produced through the decay of sulfur-containing amino acids^7^. Hydrogen sulfide (H_2_S), methyl mercaptan (CH_3_SH), and, to a lesser extent, dimethyl sulfide comprise 90% of the VSCs in halitosis^8^. VSCs have been reported to be major contributors to halitosis. Gram-negative anaerobic microorganisms, such as *Porphyromonas gingivalis*, *Treponema denticola,* and *Tannerella forsythia* contribute to increased VSC levels^9^. *Pg, Td,* and *Tf* levels are also associated with periodontal diseases^10^. To date, it has been difficult to draw clear conclusions about whether microorganisms, including bacteria and fungi, act as mediators between halitosis and periodontitis.

In clinical practice, patients with periodontal diseases often complain about bad breath. Periodontal diseases such as periodontitis and gingivitis are common, with a worldwide prevalence ranging from 20 to 50%^11^. Periodontitis is a plaque-induced chronic inflammatory disease of the supporting periodontal tissues. Gingivitis is a reactive disease that can be reversed by appropriate treatment; however, if left untreated, it can progress to a more severe form, periodontitis, which is characterized by irreversible damage to the periodontium and loss of bone or periodontal support^12^. Several clinical studies have investigated the relationship between halitosis and the development of periodontal disease^13,14^. Clinical symptoms of periodontitis may include not only oral malodor but also bleeding gums, pain, increased tooth mobility, and ultimately tooth loss^15^. Additionally, the occurrence of halitosis in patients with periodontitis is a major factor that deteriorates their quality of life^16,17^. Despite the importance of these diseases, previous studies have been limited to pilot studies, clinical studies without control groups, and reviews of related papers. Therefore, an original study investigating the relationship between halitosis and periodontal disease is needed.

The hypothesis of this observational study was that H_2_S and CH_3_SH levels in patients with gingivitis and periodontitis would be significantly higher than those in healthy controls. This study aimed to determine whether VSCs levels can be used as biomarkers to infer the existence of periodontal disease. The participants were divided into healthy control, gingivitis, and periodontitis groups by a periodontist, and their H_2_S, CH_3_SH, and VSC levels were measured using gas chromatography. Additionally, we aimed to evaluate whether sex, age, salivary flow rate, and smoking habits affect halitosis in patients with periodontal disease. To examine the relationship between halitosis and periodontal disease in depth, their incidence rates were examined, and various statistical approaches were implemented for their correlation.

## Materials and methods

### Study population

For this case–control study, healthy controls and patients with periodontal disease were recruited through in-hospital advertisements at Kyung Hee University Dental Hospital (Seoul, Korea) between May 1, 2021, and January 31, 2022. The inclusion criteria were as follows: Korean adults aged > 20 years; ≥ 20 remaining natural teeth, excluding wisdom teeth, at the time of the first examination; and healthy periodontium, gingivitis, or chronic periodontitis diagnoses (all defined below). Participants were classified based on full-mouth records of clinical parameters, including probing depth (PD), clinical attachment loss (CAL), and bleeding on probing (BOP), which were measured at six sites per tooth using a periodontal probe (Probe UNC 15; Hu-Friedy). Periodontal assessments were conducted by two experienced periodontists (JYH and SIS), and radiographic examinations were performed to confirm the diagnosis for each participant. Calibration exercises by measuring CAL in 10 patients at 24-h intervals were conducted by two trained examiners. Intra-class correlation coefficients (ICCs) of 0.80 and 0.89 were estimated in intra-examiner reproducibility measurements. Inter-examiner ICCs for CAL were 0.81 and 0.82 in each measurement. When there was disagreement, a unified conclusion was reached through several discussions until a consensus was reached.

The case definitions of healthy periodontium, gingivitis, and chronic periodontitis were based on the criteria defined in the 2017 World Workshop on the Classification of Periodontal Diseases^18^. A clinically healthy periodontium was diagnosed when the subject showed a PD ≤ 3 mm, BOP sites < 10%, and no CAL. Healthy controls were defined as individuals who did not have any major systemic diseases or were not regularly taking medications for physical or psychological conditions. Patients with periodontal disease were classified into the gingivitis and periodontitis groups. Participants with BOP sites ≥ 10% and PD ≤ 3 mm in all sites were diagnosed as gingivitis and included in the respective group (gingivitis group). The periodontitis group comprised participants with: (1) interdental CAL > 5 mm at the site of greatest loss, (2) radiographic bone loss of more than mid-third of the root, (3) ≤ 4 teeth lost because of periodontitis, (4) in addition to Stage II complexity (maximum PD ≤ 5 mm, mostly horizontal bone loss), PD ≥ 6 mm, vertical bone loss ≥ 3 mm, furcation involvement Class II or III, or moderate ridge defect, which corresponds to Stage III, or patients with (1), (2) from Stage III criteria, (3) ≥ 5 teeth lost because of periodontitis, (4) in addition to Stage IV complexity, less than 20 remaining teeth (10 opposing pairs), need for complex rehabilitation, or sever ridge defect, which corresponds to Stage IV in the generalized pattern.

The exclusion criteria were as follows: history of periodontal treatment within the last 6 months, taking medications that may affect periodontal conditions (antibiotics, anti-inflammatory drugs, anticonvulsants, immunosuppressants, calcium channel blockers, etc.) within the last 6 months, taking anticoagulants (e.g., aspirin), having uncontrolled diabetes mellitus and other systemic diseases, pregnancy or breastfeeding; and (6) the presence of intraoral appliances as part of orthodontic treatment.

For sample size calculation, we used G*Power software (ver. 3.1.9.7; Heinrich-Heine-Universität Düsseldorf, Düsseldorf, Germany), found that 89 participants (α level = 0.05, the power = 0.90, and the effect size = 0.5) with an actual target of at least 30 per group were suitable for statistical analysis, and a total of 104 participants were recruited. All participants were given information about the study and provided informed consent, and all protocols were approved by the Committee on Ethics of the Kyung Hee Clinical Research Institute, Kyung Hee University Medical Center (IRB no. KH-DT20030).

### Study design

#### (1) VSC measurement

For the participants in the validation sample, H_2_S and CH_3_SH levels in mouth air were measured using a portable gas chromatograph (TwinBreasor II, IsenLab, Gyeonggido, Korea) equipped with a flame photometric detector. As a 10 mL sample of the participant’s mouth air passed through an electrolytic sensor, the concentrations of H_2_S and CH_3_SH were detected, indicating a peak level in ppb on the digital scale of the monitor. Halitosis measurement was performed between 9:00 am and 11:00 am in a well-ventilated laboratory environment with no olfactory, visual, or auditory stimuli that interfere with accurate measurements. On the day of measurement, the participants were measured while maintaining their usual eating habits and limiting the use of alcohol, cosmetics, and perfume that could affect the VSC level. The concentrations of H_2_S and CH_3_SH and their sum (total VSCs) were expressed in parts per billion (ppb). The presence of halitosis referred to a previous study on Korean participants that sought the cut-off value for halitosis diagnosis; 65.79 ppb for women and 79.94 ppb for men were diagnostic reference point for halitosis^19^.

### (2) Salivary flow rate

Prior to the laboratory collection of saliva, the participants were instructed to refrain from caffeine and/or nicotine for at least 4 h and alcohol for at least 24 h. Saliva was collected between 9:00 am and 11:00 am to minimize circadian differences. UFR was obtained by measuring the amount of saliva collected using the spitting method for 10 min while the patient was resting. After a 2-min pre-stimulation period to remove the saliva retained in the ducts, the SFR was determined by measuring the amount of saliva collected while chewing gum for 5 min with habitual chewing of 1 g of gum base. UFR and SFR were expressed in mL/min^20^.

#### (3) Smoking habit

As for the existence of a current smoking habit, participants answered yes/no to the question, “Have you smoked cigarettes, e-cigarettes, or cigars in the last month?”.

#### (4) Statistics

Data were analyzed using the Statistical Package for the Social Sciences (SPSS) for Windows (version 26.0; IBM Corp. Armonk, NY, USA). Descriptive statistics are reported as mean ± standard deviation or numbers with percentages, where appropriate. To analyze the distribution of discontinuous data, we used the χ^2^ and Bonferroni tests for equality of proportions. Analysis of variance and Tukey’s post-hoc test was used to compare the values of the parameters among the healthy control, gingivitis, and periodontitis groups. Spearman’s correlation analysis was used to determine the relationship between H_2_S and CH_3_SH concentrations. The correlation coefficients (r) indicate the strength of the correlation and range between − 1 and 1; the closer the absolute value of r is to 1, the stronger the relationship. Multivariate logistic regression analysis was conducted to assess whether any independent variable led to a significant increase in the probability of the outcome variable (the presence of halitosis). Odds ratios (ORs) were calculated to determine how a unit change in a dependent variable affected the likelihood of the presence of an outcome variable, wherein ORs greater than 1 implied a greater relative risk. To show the performance at the classification threshold (above the mean value of each laboratory parameter), a receiver operating characteristic (ROC) curve was plotted, and the area under the ROC curve (AUC) value was calculated for each model. As the rule of thumb for interpreting the AUC value, AUC = 0.5 (no discrimination), and AUC > 0.9 (outstanding discrimination)^21^. For all analyses, statistical significance was set at a two-tailed p-value < 0.05.

### Ethics statement

The research protocol for this study complied with the Declaration of Helsinki and was approved by the Institutional Review Board of Kyung Hee University Dental Hospital in Seoul, South Korea (KHD IRB, IRB No-KH-DT20030). Informed consent was obtained from all the participants.

## Results

### Demographics

A total of 104 voluntary participants (mean age = 46.49 ± 16.03 years, 52 females and 52 males) were included in this study. Patients were divided into three groups by periodontitis experts: healthy controls, patients with gingivitis, and patients with periodontitis. The male-to-female ratio was 1:1 and there was no significant difference in sex distribution by group (p = 0.786). However, the mean age of each group was significantly higher in periodontitis (n = 33, 56.79 ± 11.70 years), followed by gingivitis (n = 43, 45.67 ± 16.44 years), and healthy controls (n = 28, 38.82 ± 14.23 yeas) (p < 0.001). Additionally, 14.3% of patients with periodontitis, 9.3% of patients with gingivitis, and 3.0% of healthy controls had a recent smoking habit, with no significant difference in the rates (p = 0.291) (Table 1). Among 28 patients with periodontitis, stage III and stage IV were 26 (92.86%) and 2 (7.14%), respectively. Since most of the periodontitis group belonged to Stage III, additional analysis by periodontitis stage was not performed.

**Table 1:** Demographics and salivary flow rate.

	Healthy control (n = 33) mean ± SD or n (%)	Gingivitis (n = 43) mean ± SD or n (%)	Periodontitis (n = 28) mean ± SD or n (%)	p-value
Demographics				
Age (years)^a^	38.82 ± 14.23	45.67 ± 16.44	56.79 ± 11.70	< 0.001***
Sex^b^				
Male	18 (54.5%)	20 (46.5%)	14 (50.5%)	0.786
Female	15 (45.5%)	23 (53.5%)	14 (50.0%)	
Smoking habit	1 (3.0%)	4 (9.3%)	4 (14.3%)	0.291
Salivary characteristics				
UFR^a^	1.12 ± 0.33	1.28 ± 0.50	1.32 ± 0.55	0.202
SFR^a^	1.61 ± 0.66	1.53 ± 0.63	1.50 ± 0.63	0.792

*SD* standard deviation, *SFR* stimulated salivary flow rate, *UFR* unstimulated salivary flow rate.

^a^Analysis of variance and Tukey’s paired comparison test were used to compare the mean values among the three groups.

^b^Results were obtained using a two-sided chi-square test (two-sided). Statistical significance was set at a p < 0.05. ***p < 0.001.

Significant values are given in bold.

### Unstimulated and stimulated saliva flow rate

In the case of UFR, there was no significant difference among the healthy control (1.12 ± 0.33 mL/min), gingivitis (1.28 ± 0.50 mL/min), and periodontitis (1.32 ± 0.55 mL/min) (p = 0.202) groups. In the case of SFR, there was no significant difference between healthy controls (1.61 ± 0.66 mL/min), gingivitis (1.53 ± 0.63 mL/min), and periodontitis (1.50 ± 0.63 mL/min) groups (p = 0.792) (Table 1).

### Volatile sulfur compounds and halitosis

**Figure 1:**
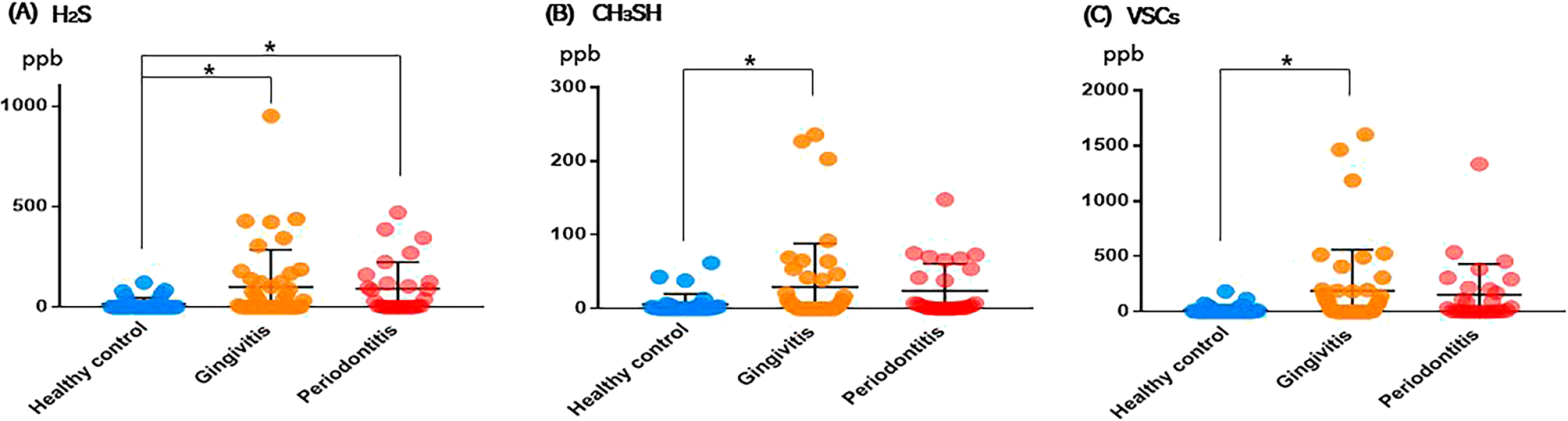
Distribution of hydrogen sulfide, methyl mercaptan, and VSCs.

Hydrogen sulfide (H_2_S) was significantly higher in the gingivitis (100.51 ± 183.69 ppb) and periodontitis (91.57 ± 132.06 ppb) groups than in healthy controls (14.97 ± 31.22 ppb) (p = 0.021), and there was no significant difference between the gingivitis and periodontitis groups. In the case of methyl mercaptan (CH_3_SH), there was a significant difference between the healthy control (5.73 ± 14.10 ppb) and gingivitis (29.31 ± 59.16 ppb) groups (p = 0.048), and there was no significant difference between the gingivitis and periodontitis groups. In the case of VSCs, it was significantly higher in gingivitis than in healthy control (186.72 ± 374.83 ppb vs. 19.80 ± 40.19 ppb, p = 0.035), and there was no significant difference between gingivitis and periodontitis (153.79 ± 278.51 ppb) groups (Fig. 1).

### Prevalence of halitosis and periodontitis and their association

When examining the rates of halitosis and non-halitosis, the rate of halitosis was significantly higher in patients with periodontal disease than in healthy controls. Halitosis was determined based on previously reported cut-off value, 65.79 ppb in female and 79.94 ppb in male; in our study halitosis was found in 3% of healthy controls, 39.5% of gingivitis, and 42.9% of periodontitis, which was significantly higher in the gingivitis and periodontitis groups than in the healthy controls (p = 0.005) (Table 2). The incidence of halitosis increased in the following order: healthy controls, patients with gingivitis, and patients with periodontitis.

**Table 2:** Volatile sulfur compound level and distribution of halitosis by group.

	Healthy control (n = 33) mean ± SD or n (%)	Gingivitis (n = 43) mean ± SD or n (%)	Periodontitis (n = 28) mean ± SD or n (%)	p-value
VSC level				
H_2_S (ppb)^a^	14.97 ± 31.22	100.51 ± 183.69	91.57 ± 132.06	0.021*
CH_3_SH (ppb)^a^	5.73 ± 14.10	29.31 ± 59.16	24.00 ± 36.98	0.048*
VSCs (ppb)^a^	19.80 ± 40.19	186.72 ± 374.83	153.79 ± 278.51	0.035*
Objective halitosis				
Non-halitosis^b^	30 (90.9%)	26 (60.5%)	16 (57.1%)	0.005**
Halitosis^b^	3 (9.1%)	17 (39.5%)	12 (42.9%)	

*SD* standard deviation, *VSCs* volatile sulfur compounds.

^a^Analysis of variance and Tukey’s paired comparison test were used to compare the mean values among the three groups.

^b^Results were obtained using a two-sided test (two-sided).

Statistical significance was set at P < 0.05. *p < 0.05, **p < 0.01. Significant values are given in bold.

**Figure 2:**
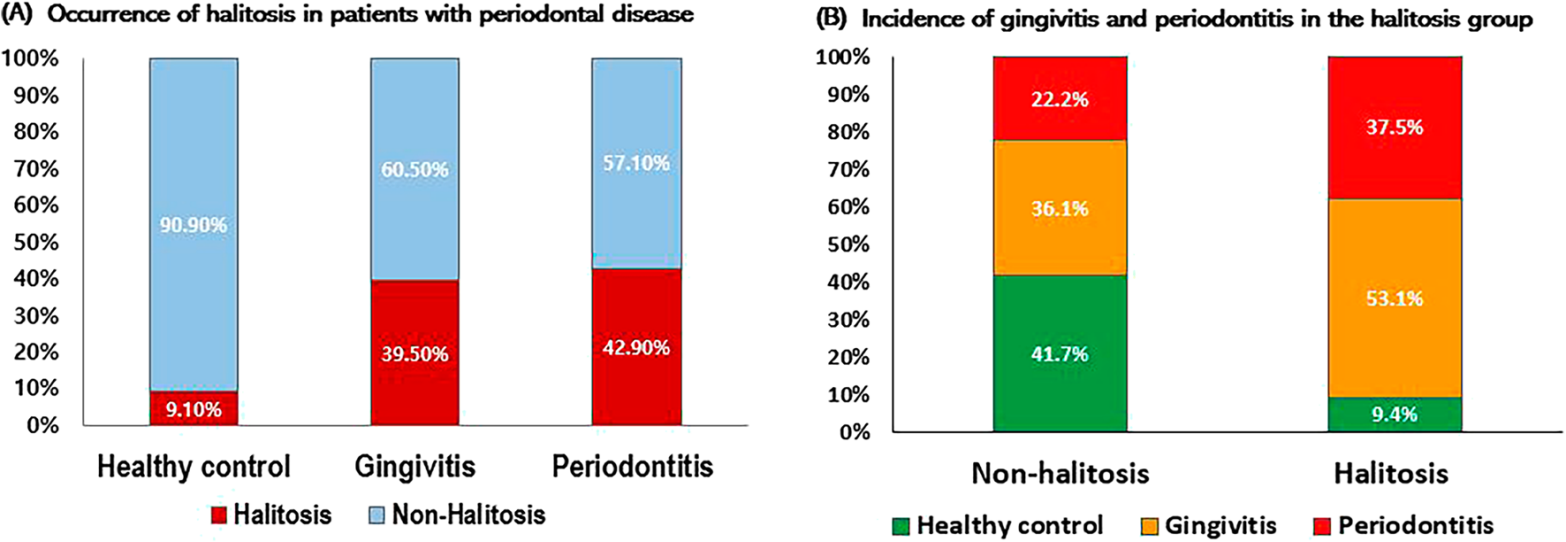
Mutual occurrence of halitosis and periodontitis.

When all participants were divided into non-halitosis and halitosis groups according to the presence or absence of halitosis according to the criteria mentioned above and the distribution of periodontal disease was examined, there was a significant difference between the groups. In the non-halitosis group, gingivitis was 36.1% and periodontitis was 22.2%, and the total incidence of periodontal disease was 58.3%. In the halitosis group, gingivitis, periodontitis, and periodontal disease accounted for 53.1%, 37.5%, and 90.6% of cases, respectively (Fig. 2). Gingivitis and periodontitis rates were significantly higher in the halitosis group than in the non-halitosis group (p = 0.005).

### Correlations among the H_2_S, CH_3_SH, and total VSCs

**Figure 3:**
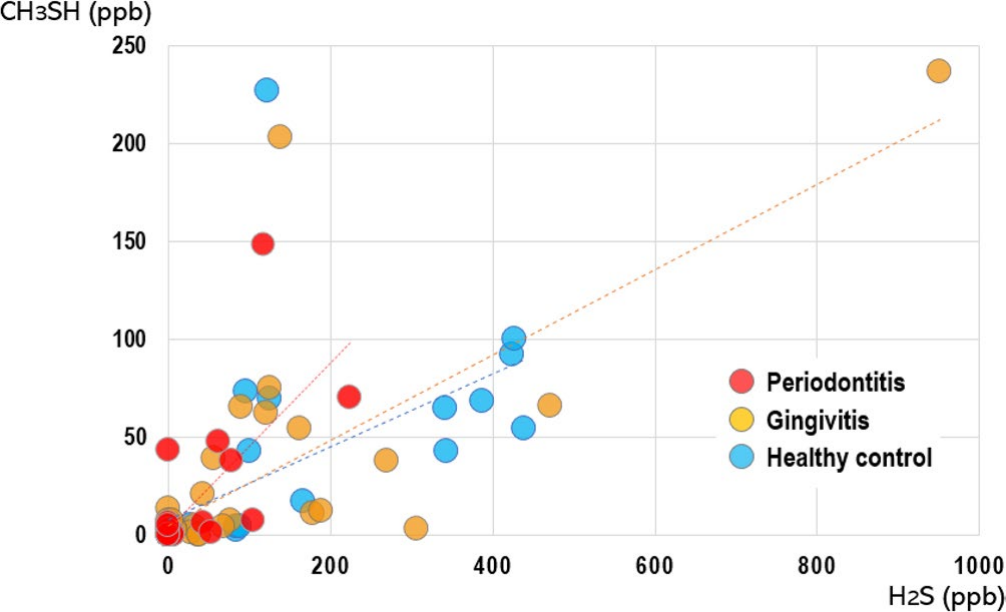
Correlation between H_2_S and CH_3_SH in patients with gingivitis and periodontitis.

To determine whether there is a correlation between H_2_S and CH_3_SH, and which factors have a stronger correlation with the increase in total VSCs, Spearman's correlation analysis was performed (Table 3). When the correlation between H_2_S and CH_3_SH was obtained by group, the gingivitis group had the highest positive correlation coefficient (r = 0.861, p < 0.001), followed by the periodontitis group (r = 0.732, p < 0.001) and healthy controls (r = 0.562, p < 0.001) (Fig. 3). The magnitude of the correlation between the increase in VSC levels and the H_2_S and CH_3_SH levels differed between healthy controls and patients with periodontal disease. In healthy controls, CH_3_SH (r = 0.900, p < 0.001) had a stronger correlation than H_2_S (r = 0.817, p < 0.001); however, in the gingivitis patient group, H_2_S had a stronger correlation with VSC than CH_3_SH. Additionally, in the periodontitis group, H_2_S had a stronger correlation with VSC than with CH_3_SH. This indicates that the gas that increases VSC differs between the healthy control and periodontal disease patient groups. Specifically, hydrogen sulfide (H_2_S) had a stronger correlation than methyl mercaptan (CH_3_SH) with total VSC level in patients with periodontal disease.

**Table 3:** Correlation between H_2_S, CH_3_SH, and total VSC levels.

		Healthy control (n = 33)		Gingivitis (n = 43)		Periodontitis (n = 28)	
		H_2_S	CH_3_SH	H_2_S	CH_3_SH	H_2_S	CH_3_SH
VSCs	Spearman's rho	0.817***	0.900***	0.959***	0.933***	0.955***	0.840***
	p-value	< 0.001	< 0.001	< 0.001	< 0.001	< 0.001	< 0.001
H_2_S	Spearman's rho		0.562***		0.861***		0.732***
	p-value		< 0.001		< 0.001		< 0.001

Spearman's rho among H_2_S, CH_3_SH, and the VSCs was obtained using Spearman’s correlation test.

*VSCs* volatile sulfur compounds.

Statistical significance was set at P < 0.05. ***p < 0.001. Significant values are given in bold.

### Multiple logistic regression analysis for halitosis

Multivariate logistic regression analysis was used to predict the presence of halitosis, with age, sex, smoking habits, unstimulated and stimulated salivary flow rates, periodontal diseases, and periodontitis as explanatory variables. The outcome variable was the presence of objective halitosis according to the criteria presented above (reference to halitosis: 65.79 ppb in female and 79.94 ppb in males). Periodontal disease was defined as gingivitis or periodontitis, and healthy controls were used as references. The healthy control and gingivitis groups were assumed to have non-periodontitis and were used as references, and whether the presence of periodontitis was also a significant predictor of halitosis was investigated. Among these explanatory variables, only periodontal disease was a statistically significant predictor of halitosis (OR = 3.607, 95% CI 1.023–12.718, p = 0.046) (Table 4). Thus, periodontal problems can be interpreted as major causes of halitosis.

**Table 4:** Multiple logistic regression analysis for the presence of halitosis.

All participants (N = 104)	OR	95% CI		p-value
Variable		Lower	Upper	
Age^a^ [ref.: < average value]	0.667	0.243	1.830	0.432
Male [ref. = female]	1.258	0.479	3.309	0.641
Smoking habit [ref. = none]	0.925	0.191	4.478	0.923
UFR^a^ [ref.: < average value]	0.954	0.308	2.954	0.935
SFR^a^ [ref.: < average value]	1.533	0.563	4.176	0.403
Periodontal disease^b^ [ref. = healthy control]	**3.607**	1.023	12.718	0.046*
Periodontitis [ref. = none]	1.309	0.452	3.790	0.620

*CI* confidence interval, *OR* odds ratio.

^a^Value of the parameter is above the average compared to when it is below the average.

^b^The presence of periodontal disease indicates having either gingivitis or periodontitis and was analyzed using a healthy control as a reference. Results were obtained using multivariate logistic regression analysis.

Statistical significance was set at p < 0.05. *p < 0.05. The significant results are indicated in bold font.

### Cut-off value of H_2_S and CH_3_SH for halitosis

**Figure 4:**
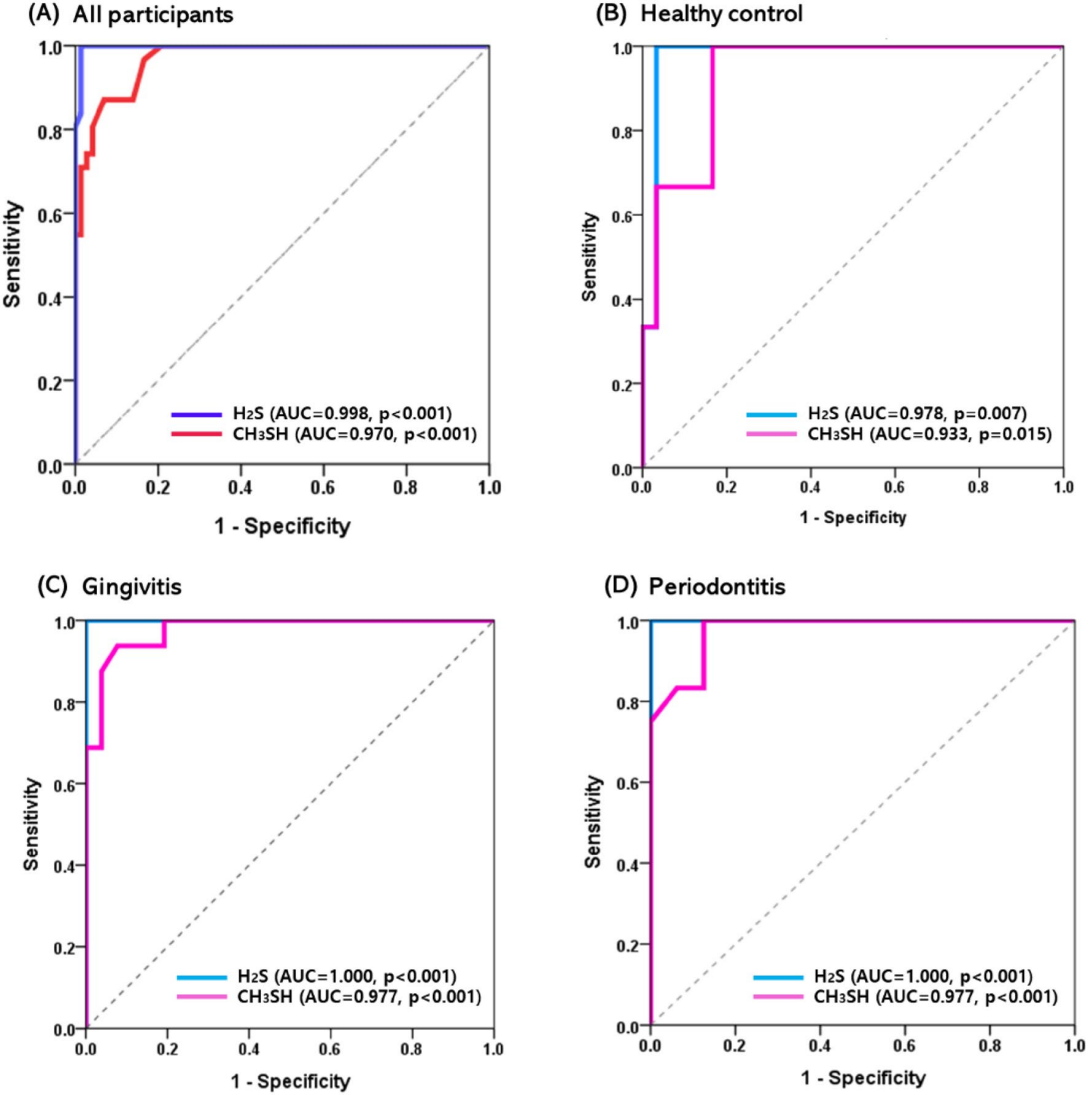
ROC curve and AUC of H_2_S and CH_3_SH to predict the halitosis. In (**A**) all participants, (**B**) healthy control, (**C**) gingivitis, and (**D**) periodontitis groups.

In all participants, the cut-off values for discrimination the presence of halitosis were 55.5 ppb for H_2_S (AUC 0.998, p < 0.001) and 6.5 ppb for CH_3_SH (AUC 0.970, p < 0.001). When analyzed by group, both H_2_S and CH_3_SH showed outstanding discrimination for halitosis, with an AUC greater than 0.93 in all three groups. In the healthy control group, H_2_S was 61.5 ppb (AUC 0.978, p < 0.007) and CH_3_SH was 3.5 ppb (AUC 0.933, p < 0.015), respectively. The gingivitis and periodontitis groups had higher AUC values for both H_2_S and CH_3_SH than the healthy controls. Particularly, for H_2_S, the AUC was 1 in both the gingivitis and periodontitis groups (Fig. 4). This result is consistent with the fact that H_2_S has a stronger correlation with the total VSC value than CH_3_SH in the correlation analysis. The cut-off values for halitosis in patients with periodontal disease were 50 ppb for H_2_S and 6 ppb for CH_3_SH in the gingivitis group, and 62 ppb for H_2_S and 3.5 ppb for CH_3_SH in the periodontitis group (all p < 0.001) (Table 5).

**Table 5:** Cut-off value for halitosis.

Prediction for halitosis		Cut-off value (ppb)	AUC	95% CI		p-value
				Upper	Lower	
All participants	H_2_S	55.5	0.998	0.992	1.000	< 0.001***
	CH_3_SH	6.5	0.970	0.943	0.997	< 0.001***
Healthy control	H_2_S	61.5	0.978	0.928	1.000	0.007**
	CH_3_SH	3.5	0.933	0.827	1.000	0.015*
Gingivitis	H_2_S	50	1.000	1.000	1.000	< 0.001***
	CH_3_SH	6	0.977	0.941	1.000	< 0.001***
Periodontitis	H_2_S	62	1.000	1.000	1.000	< 0.001***
	CH_3_SH	3.5	0.977	0.932	1.000	< 0.001***

The results were obtained using AUC analysis.

*AUC* area under the curve, *CI* confidence interval.

Statistical significance was set at p < 0.05. *p < 0.05, **p < 0.01, ***p < 0.001. The significant results are indicated in bold font.

## Discussion

This study validated our hypothesis that H_2_S and CH_3_SH levels were significantly higher in patients with gingivitis and periodontitis than in healthy controls. Additionally, periodontal disease was identified as a biomarker for the presence of halitosis based on the total VSC level; conversely, halitosis was a major predictor for the presence of periodontal disease, as demonstrated by the coexistence ratio between diseases. Interestingly, the presence of periodontitis was not a significant predictor of halitosis, rather it appeared to be a significant predictor of halitosis only when gingivitis and periodontitis were set together as parameters for periodontal disease. Moreover, clinical factors such as sex, age, salivary flow rate, and smoking habits were not significant predictors; only periodontal disease was a significant predictor of objective halitosis. This study had a prospective research design, and objective measurement of halitosis based on gas chromatography and oral examination was performed. In previous reviews on the relationship between halitosis and periodontal disease, a close association between the two diseases was inferred and suggested^14,22,23^, but prospective original research is still lacking. In this study, the incidence of halitosis was investigated according to the severity of periodontal disease, and the incidence increased in the order of healthy control (9.10%), gingivitis (39.50%), and periodontitis (42.90%). Furthermore, our results showed that periodontal disease was present in over 90% of individuals with halitosis. The high co-occurrence of periodontal disease with halitosis suggests a mutual relationship between these two diseases.

The total VSC level was significantly higher in the gingivitis than the healthy control group, and there was no significant difference between the gingivitis and periodontitis groups or between the healthy control and periodontitis groups. The major compounds that cause oral malodor include hydrogen sulfide (H_2_S), methyl mercaptan (CH_3_SH), and dimethyl sulfide (CH_3_SCH_3_), which account for approximately 90% of VSCs^24^. Increased VSC levels in the oral cavity are associated with the number and extent of periodontal pockets deeper than 3 mm^25^. Additionally, patients with periodontitis with periodontal pockets deeper than 5 mm had a 30% increase in VSC value compared to that in periodontally healthy subjects^26^. Conversely, periodontal therapies, such as pocket elimination or reduction surgery and subgingival curettage, can reduce VSC level^27^. Some researchers have suggested that the presence and severity of periodontal disease may contribute to the intensity of halitosis^28,29^. However, Bossy et al. demonstrated that oral malodor was not associated with periodontitis^30^. In a study of patients with moderate periodontitis, initial periodontal therapy—including tongue scraping—had no significant effect on the microbial load of the tongue, and only a weak effect on VSC levels^31^. Tongue coating and the oral microbiome producing VSCs of the dorsum tongue have been considered the main cause of intra-oral halitosis^32–34^. However, the causes of halitosis may differ according to age. According to Bollen et al., halitosis occurs due to tongue coating in young individuals and periodontitis with tongue coating in older individuals^6^. Even, in a cohort study investigated a population under aged 40 years, tongue coating did not mediate halitosis^35^. These confusing results are thought to be due to the different ages and/or races of the subjects and different research methodologies. Additional research is needed to draw a clear conclusion about the relationship between periodontal disease and VSC levels.

In this study, H_2_S levels were significantly higher in the gingivitis and periodontitis groups than in the healthy controls, and the AUC of H_2_S for halitosis was higher than that of CH_3_SH in all three groups. This finding suggests that H_2_S may be a useful biomarker for predicting halitosis in patients with periodontal disease. According to Persson et al., hydrogen sulfide was the predominant VSC detected in 77.2% of the periodontal pockets studied, whereas methyl mercaptan was found in approximately 20% of the pockets^36^. That is, hydrogen sulfide was detected at a higher frequency than methyl mercaptan in people with periodontal pockets. Furthermore, hydrogen sulfide and methyl mercaptan are different precursors that produce bacteria. Oral bacteria, including *Porphyromonas gingivalis, Prevotella intermedia, Fusobacterium nucleatum, Treponema denticola*, and *Veillonella alcalescens* produce hydrogen sulfide from l-cysteine^37–39^. At the genus level, *Peptostreptococcus*, *Eubacterium*, *S elenomonas*, *Centipeda*, *Bacteroides,* and *Fusobacterium* produce hydrogen sulfide. However, among VSC, methyl mercaptan is considered the main component of malodorous compounds causing periodontitis^40^. Methyl mercaptan is produced from l-methionine by a variety of microorganisms such as *P. gingivalis*, which is considered the most potent producer^41^. Since the oral microbiome was not investigated in this study, it is difficult to have a clear answer to which of the two sulfur-contained gases has a greater effect on the progression of periodontal disease and how changes in the oral microbiome lead to changes in the VSC. It is necessary to further explore the relationship between oral microflora, H_2_S, and CH_3_SH in gingivitis and the progression from gingivitis to periodontitis in periodontally healthy individuals.

Periodontal diseases include gingivitis and periodontitis. The two subtypes have different characteristics in terms of reversibility and destructiveness. Gingivitis is inflammation of the gingiva alone, and periodontitis is characterized by the destruction of the periodontium^42,43^. Although gingivitis has reversible changes that can be recovered by periodontal treatment, periodontitis leaves structural defects even after treatment and, in severe cases causes tooth loss^44,45^. In this study, periodontitis was not a significant predictor; however, periodontal disease, including gingivitis and periodontitis, was a significant predictor of halitosis. Ratcliff et al. suggested the potential importance of VSC in the transition of periodontally healthy tissue to gingivitis, and then to periodontitis^43^. Gingivitis is caused by an immune response to antigens in dental plaques and alterations in the connective tissues. VSCs can potentially alter the permeability of the gingival tissue. However, no inflammatory response was initiated by the topical application of bacterial antigens to healthy gingiva, and exposure of these tissues to H_2_S facilitated the penetration of antigens and resulted in inflammation^46^. Gingivitis is associated with the induction of an immune response with alterations in fibroblast function. CH_3_SH induces the secretion of interleukin 1-beta (IL-1β) from mononuclear cells^47^. IL-1β plays a significant role in progression in periodontal disease. CH_3_SH can act synergistically with both lipopolysaccharides and IL-1β, and increase the secretion of prostaglandin E2 and collagenase, they are important mediators of gingival inflammation and periodontal tissue destruction^48^. Both H_2_S and CH_3_SH increase the permeability of intact oral mucosa and stimulate the production of cytokines that are associated with periodontal disease. It is assumed that these VSCs are primarily important in the progression of periodontally healthy status to gingivitis, and then contribute to the development of periodontitis.

In this study, neither unstimulated nor stimulated salivary flow rates were significantly reduced in patients with gingivitis and periodontitis compared to healthy controls, and there was no significant association with halitosis. Owing to the antibacterial and self-purifying effects of saliva, it is assumed that a decrease in saliva is related to an increase in halitosis^49^. A previous study investigated the UFR and SFR in healthy subjects and compared those with mild, moderate, and severe periodontitis and observed a significant decrease in salivary flow rates with the severity of periodontitis was observed^50^. Lower salivary flow rate (under 0.28 mL/min) and smoking, as well as poor oral hygiene habits are correlated with the severity of periodontitis^51^. A large community-dwelling population study found that a lower salivary flow rate was associated with an increased risk of periodontal disease^52^. Because the present study had a prospective design and included voluntary participants, it is difficult to conclude that the clinical characteristics fully represent patients with gingivitis and periodontitis without bias. Patients who took drugs that affect the rate of salivary flow or had serious systemic diseases were excluded. Contrary to our findings, reduced salivary flow rate may be associated with halitosis^53^. Moreover, extreme reductions in salivary flow rates can be strongly associated with halitosis^54^. However, the participants in our study had a UFR of 1.24 ± 0.47 mL/min and SFR of 1.55 ± 0.621 mL/min, and systemic diseases that can affect salivary flow rate were set as a confounding or explanatory variable in other studies and not in our study, which can explain this difference in results. Because of this controversy, a large-scale, in depth study on the relationship between salivary flow rate and halitosis in patients with periodontal disease is needed.

In summary, the results of various statistical analyses showed a close relationship between periodontitis and halitosis. Halitosis was found in 3% of healthy controls, 39.5% of gingivitis patients, and 42.9% of periodontitis patients; conversely, the incidence of periodontal disease in individuals with halitosis was 90.6%. In each VSC gas, the H_2_S levels were significantly higher in the gingivitis and periodontitis groups than in the healthy controls, and the CH_3_SH level was significantly higher in the healthy control group than in the gingivitis group. The correlation coefficients between H_2_S and CH_3_SH were in the order of healthy controls < gingivitis < periodontitis groups. In a multivariate logistic regression analysis to predict the presence of halitosis, the presence of periodontal disease was a statistically significant predictor of halitosis (OR = 3.607). Two members of the VSCs family, H_2_S and CH_3_SH, are considered the primary sources of oral malodor^55,56^. Our results suggest that VSCs can be used to distinguish between gingivitis and periodontitis in preparation for healthy control. Furthermore, H_2_S levels are a major predictor of halitosis in patients with periodontal disease.

This study had some limitations. First, there was no significant difference in sex distribution between the groups, but the age of patients with gingivitis and periodontitis was significantly higher than that of healthy controls. This finding suggests the possibility of bias due to age. Clinically, patients with periodontitis are common in the 50 + age group, but it is difficult to recruit periodontally healthy controls aged 50 + years. The infectious, inflammatory, and immune features of periodontitis should be evaluated after reflecting many related systemic diseases such as cardiovascular disease and diabetes mellitus^57,58^. Since this study excluded individuals with uncontrolled systemic diseases, further studies are needed to clarify the effect of systemic disease on halitosis in patients with periodontal diseases. This study has the strength of identifying gingivitis, periodontitis, and normal groups by periodontal experts, and objective VSC measurements were performed using gas chromatography. However, investigations of oral microorganisms that cause VSCs production have not been conducted using gas chromatography to measure VSCs. Therefore, further investigation of the oral microbiome in each oral region of these patients is needed, especially in the tongue and subgingival pockets. Additionally, it is necessary to clearly support our conclusion by examining the relationship between each pathogen and VSC levels and the mutual influence between them in the periodontal disease group.

## Data Availability

The datasets used and/or analysed during the current study available from the corresponding author on reasonable request.
